# Folic Acid–Conjugated PLGA Nanoparticles of Eugenol: Development, Characterization, and In Vitro Cytotoxicity Studies on Breast Cancer Cell Line

**DOI:** 10.1155/bmri/2898012

**Published:** 2026-04-27

**Authors:** Bhawna Kashyap, Azhar Khan, Tabarak Malik, Deepak N. Kapoor

**Affiliations:** ^1^ Faculty of Applied Sciences and Biotechnology, Shoolini University, Solan, Himachal Pradesh, India, shooliniuniversity.com; ^2^ Department of Biomedical Sciences, Institute of Health, Jimma University, Jimma, Ethiopia, ju.edu.et; ^3^ Division of Research & Development, Lovely Professional University, Phagwara, Punjab, India, lpu.in; ^4^ School of Pharmaceutical Sciences, Shoolini University, Solan, Himachal Pradesh, India, shooliniuniversity.com; ^5^ Chandigarh Group of Colleges Jhanjeri, Mohali, Chandigarh Pharmacy College, Mohali, Punjab, India

## Abstract

**Background:**

The primary goal of the study was to develop folic acid–conjugated eugenol‐loaded PLGA nanoparticles for the treatment of breast cancer. Eugenol is reported to have potent anticancer activity. Entrapment of eugenol in folic acid–conjugated polymeric nanoparticles is expected to enhance its availability at the cancer site and improve overall efficacy in breast cancer treatment.

**Method:**

Eugenol was isolated by the column chromatography technique. The isolated bioactive fraction was characterized by IR and NMR analyses. Polymeric NPs were prepared by the solvent emulsification–diffusion method and conjugated with FA by the EDC coupling method. The in vitro release profile for FA‐conjugated eugenol‐loaded PLGA NPs was evaluated by the dialysis membrane technique. Further in vitro anti‐inflammatory activity, in vitro antioxidant assays, and cytotoxicity studies were carried out.

**Results:**

NPs exhibiting particle size ranging from 444.2 to 928.3 nm, zeta potential ranging from −32.0 to −37.7 mV, entrapment efficiency ranging from 76.97% to 87.51%, and percent conjugation were found to be 72.59%–79.68%. The in vitro drug release profiles of the formulations were most effectively described by the Higuchi kinetic model, indicating that the mechanism governing drug release is primarily attributed to diffusion. The FTIR study indicated that there is no chemical modification of the drug, confirming its compatibility with other excipients. The morphology of the NPs was analyzed by FESEM analysis. The particulate nature of NPs showed homogenous, spherical shapes of NPs. The cell cytotoxicity studies on MDA‐MB‐231 cell lines exhibited enhanced cytotoxicity of the NPs.

**Conclusion:**

In conclusion, it was found that FA‐conjugated PLGA NPs can be a suitable platform for the targeted administration of eugenol for BC treatment.

## 1. Introduction

Breast cancer (BC) incidence is incrementally rising, positioning it as the predominant cause of cancer‐related mortality among women [1]. Due to its widespread occurrence and the varied ways it responds to different treatments and outcomes, there is a compelling need to explore natural and synthetic agents that can prevent cancer [2]. Triple‐negative breast cancer (TNBC) comprises roughly 15%–20% of all BCs and is characterized by the lack of certain proteins, namely, estrogen receptor, progesterone receptor, and human epidermal growth factor receptor‐2 expression, which are identified using immunohistochemical markers [3, 4]. TNBC exhibits a strong metastatic potential, accelerated proliferation, and an unfavorable prognosis in comparison to other subtypes of BC [5]. Because of these aggressive attributes and the absence of precise molecular therapeutic targets, conventional chemotherapies continue to serve as the established treatment for patients with TNBC [6]. However, due to the number of severe side effects, tendency toward relapse, and higher mortality rate, various phytoconstituents are also explored for the treatment of cancer.

Eugenol, an organic compound, is naturally occurring in clove oil and various spices like cinnamon, basil, and bay leaves [7]. Eugenol has been reported to have various physiological activities and several physiological functions, such as anti‐inflammatory, antioxidant, and antibacterial properties [8]. Moreover, eugenol demonstrates effects on the proliferation and metastasis of numerous tumors. For instance, it demonstrates the ability to impede the proliferation of malignant melanoma cells and trigger apoptosis [7]. Several studies have documented eugenol′s inhibitory effect on BC [9], cervical cancer [10], and prostate cancer [11].

Chronic inflammation and oxidative stress are key contributors to carcinogenesis and tumor progression. Persistent inflammation facilitates the release of proinflammatory cytokines and growth factors that promote angiogenesis and metastasis [12]. While many chemotherapeutic drugs exert their effects by inducing oxidative stress, eugenol′s mechanism of cytotoxicity appears to involve alternative pathways, despite its antioxidant properties. Simultaneously, oxidative stress caused by an imbalance between reactive oxygen species (ROS) and antioxidants leads to DNA damage, lipid peroxidation, and protein modification, fostering mutagenesis and cancer development [13]. Eugenol′s anti‐inflammatory activity mitigates the inflammatory response by modulating pathways like NF‐ĸB and COX‐2, which are often upregulated in cancer [14]. Its antioxidant activity scavenges free radicals, protecting cells from oxidative damage while sensitizing cancer cells to apoptosis. These anti‐inflammatory and antioxidant properties synergize to enhance eugenol′s cytotoxic effects, making it a promising compound for cancer therapy [15].

Studies have demonstrated that eugenol induces apoptosis in cancer cells primarily through mitochondrial dysfunction. A study reported that eugenol disrupts mitochondrial membrane potential, leading to the release of cytochrome c and activation of the caspase cascade, which triggers programmed cell death. In addition to apoptosis, eugenol has been found to arrest the cell cycle. It was observed that eugenol downregulates cyclins and cyclin‐dependent kinases, resulting in cell cycle arrest in cancer cells, thereby inhibiting proliferation [16]. Furthermore, eugenol modulates key oncogenic signaling pathways. Al‐Sharif et al. highlighted its inhibitory effects on the NF‐ĸB pathway, which is critical for cell survival and proliferation. Eugenol has been shown to modulate the Wnt/*β*‐catenin and P13K/Akt signaling pathways, both of which are associated with cancer cell growth and survival [17]. A study by Chaieb et al. revealed that eugenol exhibits cytotoxic effects through oxidative DNA damage in cancer cells, despite its antioxidant properties, suggesting a dual role depending on the cellular context [18]. The cytotoxic mechanism of eugenol is mediated through mitochondrial dysfunction, modulation of apoptotic regulators, cell cycle arrest, and inhibition of survival signaling pathways.

Folate is a crucial micronutrient integral to one‐carbon metabolic reactions, cell division, and growth [19]. The extensive utilization of folic acid (FA) conjugation is pivotal in advancing targeted anticancer drug delivery systems, specifically directed toward the FR*α* receptor [20, 21]. Reportedly, in a rat model, FA‐conjugated graphene oxide with methyl acetate–loaded paclitaxel has been shown to inhibit the proliferation of MDA‐MB‐231 cells [22]. Targeted delivery to the FR*α* receptor using FA‐conjugated small interfering RNA (siRNA) has been successfully accomplished across various cancer types, including breast, ovarian, lung, and kidney cells [23]. Hence, the current study hypothesized that eugenol‐loaded poly(D,L‐lactide‐co‐glycolide) (PLGA) nanoparticles (NPs) conjugated with FA could be an effective approach for BC treatment. For pharmaceutical and biological applications, the US Food and Drug Administration has granted approval for PLGA [24]. The biocompatibility, biodegradability, and controlled dispersion features of the PLGA polymer are of tremendous interest to the biomedical area [24].

Eugenol was selected as a core compound due to its potent anticancer effects, biocompatibility, and sustainability for NP‐based delivery. The encapsulation of eugenol within PLGA NPs, conjugated with FA for targeted delivery, enhances its therapeutic potential by ensuring precise delivery to BC cells overexpressing folate receptors. This targeted approach not only improves the efficacy of eugenol but also minimizes its potential side effects on healthy tissues, making it a promising strategy for effective BC treatment. Despite well‐documented bioactive properties of eugenol, its clinical application in cancer therapy is hindered by poor bioavailability, rapid metabolism, and nonspecific distribution. Conventional drug delivery systems fail to provide sustained and targeted delivery of eugenol to BC cells, limiting its therapeutic efficacy. While folate receptor (FA) targeting has been widely explored for synthetic chemotherapeutic drugs, its potential in enhancing the delivery of natural anticancer agents like eugenol remains underexplored. There is a lack of efficient polymeric NP‐based approaches specifically designed for the targeted delivery of eugenol to FA receptor BC cells. Thus, the present study involves conjugation of FA to the surface of eugenol‐loaded PLGA NPs with an aim to enhance the targeting and uptake of the NPs by cancer cells that overexpress folate receptors. It is expected to improve the efficacy of eugenol against BC cells.

## 2. Materials and Methods

### 2.1. Chemicals

PLGA (Mw, 50,000–75,000 Da) with a 50:50 ratio was purchased from Nomisma Healthcare Pvt. Ltd, Gujarat, India. Poly(vinyl alcohol) (PVA; Mw 85,000–124,000 Da) and dichloromethane (DCM) were procured from Sigma‐Aldrich. MDA‐MB‐231 cell lines were obtained from NCCS, Pune, India.

### 2.2. Extraction Procedure

The parts of *Syzygium aromaticum* (buds) were used for the preparation of the plant extract. *S. aromaticum* buds underwent surface sterilization employing 0.1% HgCl_2_, followed by an 8–10 min wash with triple‐distilled water. Subsequently, they were dried at room temperature for 1–2 weeks. Dried buds were ground to make fine powder. The powder was subjected to extraction using DCM:methanol (1:1 ratio) and kept in an orbital shaker at a temperature of 40°C for the next 48 h. After 48 h, the sample underwent filtration using Whatman No. 1 filter paper to obtain the crude extract. Solvent evaporation was done by a rotary evaporator [25]. After the evaporation process, the plant extract was stored in a refrigerator at 4°C for further analysis.

### 2.3. Fractionation of the Extracts

The plant extract of *S. aromaticum* was transferred into a separating funnel. For each fraction, 100 mL × 3 times of plant extract of *S. aromaticum* was taken in a separating funnel, monitored by mild shaking n‐hexane fraction was collected. A similar process of fractionation was repeated to obtain the fraction of different solvents like ethyl acetate and methanol on the basis of the polarity of the solvents [26]. After the fractionation, the fractions were dried in an oven and stored in a refrigerator at 4°C for further analysis.

### 2.4. Isolation of Pure Compound

Isolation of pure compound eugenol from *S. aromaticum* fraction was done by column chromatography technique using silica gel with a mesh size of 60–120. A slurry of preactivated silica gel was prepared by dissolving 1 g of fraction in DCM:methanol (1:1 ratio) and then dried. The column was vertically positioned on a stand and filled halfway with silica gel. Subsequently, a slurry was carefully poured into the upper portion, allowed to settle gently, and capped with absorbent cotton. The column was washed with 100 mL of petroleum ether to remove the impurities. After washing the column, the gradient elution technique was employed to run the column. One hundred milliliters of the mobile phase, petroleum ether:ethyl acetate in a 20:80 ratio, was added. The collected fractions in test tubes were marked accordingly. These marked fractions underwent thin‐layer chromatography (TLC) to assess the consistency across the various fractions.

Elution of the compound in the column by using ethyl acetate:petroleum ether fractions yielded a pale yellowish‐colored residue. The presence of eugenol in the fraction was confirmed by TLC [27].

### 2.5. Characterization of Isolated Pure Compound

The pure compound eugenol isolated from *S. aromaticum* plant was characterized by TLC, Fourier transform infrared (FTIR), nuclear magnetic resonance (NMR) (^13^C NMR and ^1^H NMR), and high‐performance thin‐layer chromatography (HPTLC) analysis.

#### 2.5.1. TLC of Isolated Pure Compound

TLC was used to confirm the presence of the compound in the isolated fraction. TLC of the isolated fraction was performed by using a mobile phase of petroleum ether:ethyl acetate (20%:80%). The plate was air‐dried and visualized in a TLC chamber. The retention factor was determined by employing the subsequent formula:

Rf=Distance travelled by the compoundDistance travelled by the solvent

#### 2.5.2. HPTLC Analysis of Pure Isolated Compound

The analysis was conducted using a silica gel‐coated aluminum plate (10 × 10 cm; Linomat 5). Before the study, the plates were prewashed and subsequently activated at a temperature of 60°C for 5 min. A consistent application of samples was ensured using a microliter syringe. The optimized mobile phase (20 mL) that was used for analysis consisted of toluene:ethyl acetate:glacial acetic acid (7:3:0.1 *v*/*v*). The linear ascending procedure was conducted within a presaturated glass chamber measuring 10 × 10 cm. Achieving high‐resolution HPTLC involved the plate undergoing dual development phases with the mobile phase. Saturation of the chamber was maintained for 30 min at room temperature (25^°^C ± 2^°^C) [28].

#### 2.5.3. FTIR Spectroscopy of Isolated Pure Compound

The investigation of eugenol′s functional groups was carried out using the Agilent Cary 630 Series FTIR spectrophotometer. To acquire FTIR spectra, a 10 mg sample was positioned on the mirror stage, and the lens was adjusted securely. After each spectrum, the lens and the stage mirror were wiped with acetonitrile to clean up the residue of the sample [29].

#### 2.5.4. NMR Analysis of Isolated Pure Compound

^1^H NMR and ^13^C NMR spectra were acquired using a cryomagnet spectrophotometer (Bruker) at 400 and 100 MHz, respectively. CDCl_3_ was used as the standard, and its singlet was assigned at *δ* 0.0 ppm [30, 31].

#### 2.5.5. High‐Performance Liquid Chromatography (HPLC) Conditions for Analysis of Eugenol

Quantification of eugenol was done by using HPLC with a ZORBAX Extend C‐18 (4.6 × 150 mm) column. The detector used was a PDA detector. The analyte was eluted using isocratic elution in a mobile phase, comprising acetonitrile and water in a 60:40 (*v*/*v*) ratio at a flow rate of 0.6 mL/min. The volume of injection was 10 *μ*L at 27°C at a detection wavelength of 280 nm, and the run time was set at 10 min [32].

### 2.6. Preparation of Eugenol‐Loaded PLGA NPs

Eugenol‐loaded PLGA NPs were synthesized by a solvent–emulsification approach [33] with some modifications. Drug (350 mg) and PLGA (350, 700, and 1050 mg) were dissolved in 10 mL of DCM (organic phase) in different ratios (1:1, 1:2, and 1:3). Three different formulations of eugenol and PLGA were prepared. Meanwhile, 1% *w*/*v* PVA (400 mg) was solubilized in distilled water (40 mL) (aqueous phase). The addition of the organic phase, which held the drug and polymer, was done gradually, drop by drop, into the aqueous phase while submerged in an ice‐cool bath and subjected to sonication for a duration of 20 min. After sonication, the sample was stirred overnight. The resulting oil‐in‐water emulsion was diluted by adding distilled water while simultaneously subjected to magnetic stirring, facilitating the dispersion of the solvent. After stirring, the sample underwent centrifugation at 12,000 rpm for 30 min. The resultant pellet was collected, and the supernatant was discarded. This pellet was weighed and redispersed in cold DW, forming a resulting nanosuspension. This nanosuspension was lyophilized and subsequently stored in an airtight bottle at 4°C [33].

#### 2.6.1. Entrapment Efficiency (EE%)

The EE% of eugenol was assessed by determining the eugenol entrapped into the NPs. NPs (10 mg) were dissolved in acetonitrile (5 mL) and sonicated for 20 min. The sample was filtered using a 0.45 *μ*m nylon syringe and analyzed for drug content by HPLC [34]. The entrapped drug content in the NPs was determined by employing the subsequent equation:

(1) EE%=Amount of drug foundAmount of drug loaded∗100

#### 2.6.2. Size Distribution and Zeta Potential Analysis

Particle size distribution of eugenol‐loaded PLGA NPs was carried out by dynamic light scattering (DLS) (Malvern Zetasizer S90 series). The samples were resuspended in Milli‐Q water. The determination of zeta potential was conducted using DLS [35].

#### 2.6.3. FA Conjugation on the Drug‐Encapsulated NPs

The 1‐ethyl‐3‐(3‐dimethylaminopropyl)carbodiimide (EDC) coupling method was employed for the conjugation of FA with the eugenol‐loaded PLGA NPs. FA (60 mg) was dissolved in phosphate buffer (2 mL), pH 9.0, containing EDC, serving as a carboxylic group activator. The activated FA solution was introduced into a 10 mg/2 mL solution of eugenol‐loaded NPs in phosphate buffer (pH 9.0). The resulting reaction mixture underwent stirring for 3 h, after which ultracentrifugation was performed to separate the NPs′ carrier from the solution. The pellet obtained from ultracentrifugation was lyophilized and redispersed in cold DW and stored in a refrigerator for further analysis [36]. The percentage of FA conjugation was assessed by using UV/Vis spectrophotometric analysis at a wavelength of 363 nm, which is the maximum absorbance peak of FA.

#### 2.6.4. FTIR Spectroscopy Studies

The Agilent Cary 630 Series FTIR spectrophotometer was used to study the confirmation of FA conjugation in eugenol‐loaded PLGA NPs. In order to obtain FTIR spectra, a 10 mg sample of eugenol (pure drug), FA, eugenol‐loaded FA‐conjugated NPs, and a blank formulation was placed on the mirror stage, and the lens was tightened up. After each spectrum, the lens and the stage mirror were wiped with acetonitrile to clean up the residue of the sample [28].

#### 2.6.5. In Vitro Drug Release Studies of FA‐Conjugated Eugenol‐Loaded PLGA NPs

The in vitro drug release study of eugenol from different formulations of FA‐conjugated eugenol‐loaded PLGA NPs was studied by the dialysis bag diffusion method [37]. Drug‐loaded NPs (equivalent to 50 mg eugenol dispersed in 5 mL pH 7.4 phosphate buffer) were packed into a dialysis bag, subsequently placed in a beaker containing 100 mL of phosphate buffer (pH 7.4). The beaker was positioned over a magnetic stirrer, ensuring the maintenance of a constant temperature at 37°C ± 1°C throughout the duration of the experiment. Stirring was carried out at 1000 rpm. At predetermined intervals, a sample (1 mL) was taken at 15 min, 30 min, 1 h, 2 h, 4 h, 8 h, 12 h, and 24 h and substituted with an equivalent volume of fresh phosphate buffer (pH 7.4). Following appropriate dilutions, samples underwent analysis employing HPLC.

#### 2.6.6. Kinetic Modeling of the Drug Release of FA‐Conjugated Eugenol‐Loaded PLGA NPs

The drug release data collected over time underwent fitting with multiple kinetic models to ascertain the release mechanism′s nature and kinetics. Zero‐order rate (Equation 2) characterizes systems in which the drug release rate persists unaffected by its concentration [38]. First‐order (Equation 3) represents the release from a system where the drug release rate relies upon its concentration [39]. Higuchi [40] formulated the drug release from insoluble matrices as a time‐dependent process, proportional to the square root of time, based on Fickian diffusion (Equation 4). Hixon–Crowell (Equation 5) was used to describe powder dissolution or drug release from formulations, and Korsmeyer et al. derived a simple mathematical relationship (Equation 6) depicting the drug release from a polymeric system [41].

(2) Q=kt+Q0

(3) Q=Q0.ekt

(4) Q=k.t0.5

(5) Q13013/=k.t+Q /

(6) Q=k.tn

where *Q* symbolizes the cumulative quantity of active released drug at time *t*, *Q*0 represents the initial drug loading in the formulation, *k* denotes the release constant, and *n* signifies the release exponent characterizing the drug release mechanism.

#### 2.6.7. Field Emission Scanning Electron Microscope (FESEM) Analysis

The prepared FA‐conjugated eugenol‐loaded PLGA NPs were analyzed by FESEM. A small quantity of NPs was affixed onto a carbon‐coated copper grid. The sample fixed on the grid underwent a drying period of 10–15 min prior to FESEM imaging.

### 2.7. In Vitro Anti‐Inflammatory Activity of FA‐Conjugated Eugenol‐Loaded PLGA NPs

The assessment of in vitro anti‐inflammatory activity of pure drug eugenol and FA‐conjugated eugenol‐loaded PLGA NPs was studied by using the BSA (bovine serum albumin) assay [42]. BSA (0.2%) solution was prepared in Tris buffer (pH 6.8). A stock solution of eugenol and FA‐conjugated eugenol‐loaded PLGA NPs was prepared in methanol (500 *μ*g/mL). The test tubes containing the aliquots of various concentrations were taken, and 1 mL of BSA solution was added. Eugenol and NPs equivalent to eugenol concentrations of 5.0–50 *μ*g/mL were prepared. A similar test was performed on the positive (diclofenac sodium) and negative control (DMSO) and pure drug eugenol. FA‐conjugated eugenol‐loaded PLGA NPs, eugenol, and control samples underwent incubation in a water bath set at 72°C for a duration of 15 min. Further, test tubes were allowed to cool down for 30 min. Subsequently, the measurements of absorbance were conducted at a wavelength of 660 nm. The entire experiment was performed in triplicate. The subsequent equation was utilized for determining the percentage inhibition of proteins [43]:

(7) %Anti‐Denaturation activity=Absorbance of control−Absorbance of SampleAbsorbance of control ×100

### 2.8. In Vitro Antioxidant Assay of FA‐Conjugated Eugenol‐Loaded PLGA NPs

The antioxidant capacity was determined by the DPPH (2,2‐diphenyl‐1‐picrylhydrazyl) radical scavenging assay. The assessment of the free radical scavenging activity for both eugenol and eugenol‐loaded PLGA NPs was performed utilizing a DPPH solution (0.004% *w*/*v*) prepared in methanol (95%). The FA‐conjugated eugenol‐loaded PLGA NPs and eugenol were subsequently combined with methanol (95%) to prepare the stock solution with a concentration of 5 mg/mL. Freshly prepared DPPH solution was placed in test tubes, and the samples and aliquots of different concentrations were prepared. The concentration of NPs equivalent to eugenol was serially diluted in concentrations from 20 to 100 *μ*g/mL, respectively, to make up the final volume of 1 mL. After an incubation period of 20 min at ambient temperature, the samples were assessed for absorbance at 517 nm. Ascorbic acid served as the reference standard in this experiment. Methanol (95%) was served as a blank [44, 45]. The determination of the DPPH inhibition percentage was accomplished using the following equation:

(8) %DPPH inhibition=OD Control−OD SampleOD Sample∗100

### 2.9. Cytotoxicity Studies of FA‐Conjugated Eugenol‐Loaded PLGA NPs

The cytotoxicity investigation was conducted by employing the MTT assay. The cell viability of pure drug eugenol, eugenol‐loaded PLGA NPs, and FA‐conjugated eugenol‐loaded PLGA NPs was observed. MDA‐MB‐231 cells were seeded in a 96‐well microplate at a density of 7000 cells per well. The cells were incubated at 37°C in a 5% CO_2_ incubator and 98% humidity for 24 h. Following a 24‐h incubation period, the media was removed, and the cells underwent washing with 1X PBS. The treatment was given to cells at final concentrations of 3.6–1000 *μ*g/mL, equivalent to eugenol, respectively. Following a 24‐h incubation period, a mixture containing 5 *μ*g/mL of MTT solution in PBS was introduced into the wells. The plates underwent an additional incubation period of 3 h to allow for the development of formazan crystals. Subsequently, the formed formazan crystals were dissolved in DMSO (100 *μ*L). Untreated cells were kept as a control. The measurement of absorbance at 570 nm was conducted using a microplate reader (POLARstar OPTIMA, BMG Labtech, Germany). All experiments were conducted in triplicate. The cell viability was quantified using the following equation:

(9) %cell viability=Average absorbance of treatedAverage absorbance of control ∗100

### 2.10. Statistical Analysis

Data were analyzed by using ANOVA analysis, and mean values of data were compared by a post hoc analysis using Bonferroni′s multiple tests using GraphPad Prism 5.0. Statistical significance was determined at a *p* value < 0.05, indicating significant differences, while a *p* value < 0.05 denoted nonsignificant differences.

## 3. Results

The pure compound eugenol was obtained through the column chromatography technique. The isolated compound (eugenol) was characterized by TLC, FTIR, ^1^H NMR, ^13^C NMR, and HPTLC. TLC was carried out on an aluminum silica plate. TLC of the pure compound showed a single band in the mobile phase of ethyl acetate:petroleum ether in the ratio of 20%:80% with the following Rf value: 0.56 ± 0.03. The yellowish color spot in the TLC plate indicates the presence of eugenol.

The FTIR spectrum of the isolated eugenol was recorded to determine the characteristic functional groups. Figure S1 shows the spectra of the isolated compound eugenol scanned at 4000–4500 cm^−1^. The infrared‐induced vibrational frequencies of the different functional groups present in the isolated compound eugenol were recoded. The sharp peaks at 1514 and 1614 cm^−1^ were in good agreement with eugenol. The results indicated the presence of characteristic peaks of eugenol supported by the previous studies, confirming the identification of the isolated compound eugenol [46].

The structure elucidation was carried out by using NMR analysis. ^1^H NMR and ^13^C NMR were done by using a CDCl_3_ solvent on a Bruker Advance Neo 500 NMR spectrophotometer at SAIF, PU (Chandigarh). The chemical shift values of ^1^H NMR and ^13^C NMR confirmed the structure of the eugenol compound. The ^1^H NMR spectrum of the compound showed the chemical shift of protons and functional group *δ*, CDCl_3_ (500 Hz) 3.15 (2H, d, *J* = 1.5), 5.95 (1H, m), 5.05 (2H, m), 6.84 (1H, d, *J* = 4.15), 6.89 (2H, m), and 3.871 (3H, s). The presence of three protons at *δ* 5.95, 6.84, and 6.89 ppm indicated the aromatic proton in the molecule shown in Figure S2A. A methoxy group was present on the aromatic ring. The ^13^C NMR spectrum of the compound showed the chemical shift of protons and carbon groups *δ* (125 Hz) 146.44, 137.83, 131.94, 121.19, 114.25, and 55.87 as shown in Figure S2B. The chemical shift (*δ*) values of ^1^H NMR and ^13^C NMR spectra confirmed the structure of the pure compound eugenol and showed that all the above facts are in good agreement with the unique structure of eugenol, as shown in Table 1. The confirmation of eugenol′s structure via NMR is depicted in Figure S2.

**Table 1:** NMR interpretation of pure isolated compound eugenol.

Compound	Signal group	^13^C NMR interpretation (*δ* ppm), 125 Hz	^1^H NMR interpretation (*δ* ppm), 500 Hz
Eugenol	OH–H	131.94	3.323 (2H, d, *J* = 1.5)
	O–CH_3_	55.87	3.871 (3H, s)
	CH_2_	137.83	5.057 (2H, m)
	H	121.19	5.95 (1H, m)
	H	146.44	6.84 (1H, d, *J* = 4.15)
	H	114.25	6.89 (2H, m)

*Note:* *J*, coupling constant. The chemical shifts (*δ*) for both ^1^H NMR and ^13^C NMR were reported in parts per million.

Abbreviations: d, doublet; m, multiplet; s, singlet.

HPTLC analysis was done to check the percent purity of the isolated compound eugenol in comparison to pure eugenol (standard). The purity of the isolated compound eugenol was found to be 89.72*%* ± 1.18*%* by using a standard calibration curve. The HPTLC chromatogram is shown in Figure S3.

The results of the prepared eugenol‐loaded PLGA NPs with different drug:polymer ratios are shown in Table 2. Eugenol‐loaded PLGA NPs were synthesized by the modified solvent‐emulsification diffusion technique. EE% of eugenol‐loaded PLGA NPs (Table 3) were found to be in the range of 76.97*%* ± 1.09*%*–87.51*%* ± 1.47*%*. The entrapment of *F*1 formulation (1:1 ratio) was found to be 76.97*%* ± 1.09*%*, *F*2 formulation (1:2 ratio) showed EE% 78.66*%* ± 1.14*%*, and *F*3 formulation (1:3 ratio) showed EE% 87.51*%* ± 1.47*%*. The *F*3 formulation drug:polymer ratio (1:3) showed the highest entrapment of drug among all three different formulations. This could be due to the increased ratio of polymer in the formulation. With an increased PLGA polymer ratio, there is more polymer available to encapsulate eugenol.

**Figure 1:**
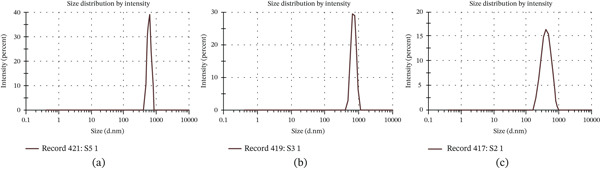
Particle size distribution of eugenol‐loaded NP formulations: (a) F1 (1:1), (b) F2 (1:2), and (c) F3 (1:3).

The formulation′s PDI (polydispersity index) was in the range of 0.345–0.524. The particle size data clearly indicate that eugenol‐loaded PLGA NPs, mean particle size of *F*1 formulation (1:1 ratio), mean particle size of 928.3 nm, and PDI were found to be 0.509, as shown in Figure 1a. For the *F*2 formulation (1:2 ratio), the mean particle size was 834.9 nm and PDI of 0.345 nm, as shown in Figure 1b. The mean particle size of the *F*3 formulation (1:3 ratio) was 444.2 nm, and the PDI was 0.338 nm, as shown in Figure 1c. From the results, it was found that all the formulations of NPs showed particles in the nanosize range with an acceptable PDI value.

**Table 2:** Particle size distribution, PDI, and zeta potential of the NPs obtained by the solvent emulsification technique. Values (*n* = 3) are mean ± SD.

Formulations	Drug:polymer	Particles size (nm)	PDI	Zeta potential (mV)
F1	1:1	928.3	0.509	−32.0
F2	1:2	834.9	0.345	−34.2
F3	1:3	444.2	0.338	−37.7

**Figure 2:**
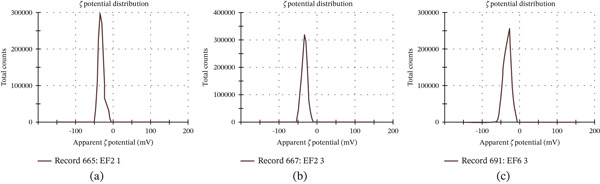
Zeta potential of eugenol‐loaded NP formulations: (a) F1 (1:1), (b) F2 (1:2), and (c) F3 (1:3).

The assessment of the formulated NPs′ stability was evaluated by analyzing the zeta potential of the synthesized particles. Zeta potential values of different formulations, which were in the range from −32.0 to −37.7 mV, as shown in Table 2, indicate that the formulations were stable. The zeta potential of formulation *F*1 (1:1 ratio) was found to be −32.0 mV, as shown in Figure 2a, whereas the zeta potential of *F*2 formulation was found to be −34.2 mV, as shown in Figure 2b, and the zeta potential of *F*3 (1:3 ratio) was found to be −37.7 mV, as shown in Figure 2c.

The percentage conjugation of FA in the formulation *F*1 was found to be 79.68*%* ± 1.30*%*; in formulation *F*2, it was found to be 75.35*%* ± 2.13*%*; whereas in formulation F3, it was found to be 72.59% ±1.30%, as shown in Table 3.

**Table 3:** Entrapment efficiency and FA conjugation percentage of the eugenol‐loaded PLGA NPs.

S. no.	Formulations	Drug:polymer	EE%	Conjugation %
1.	F1	1:1	76.97 ± 1.09	79.68 ± 1.96
2.	F2	1:2	78.66 ± 1.14	75.35 ± 2.13
3.	F3	1:3	87.51 ± 1.47	72.59 ± 1.30

**Figure 3:**
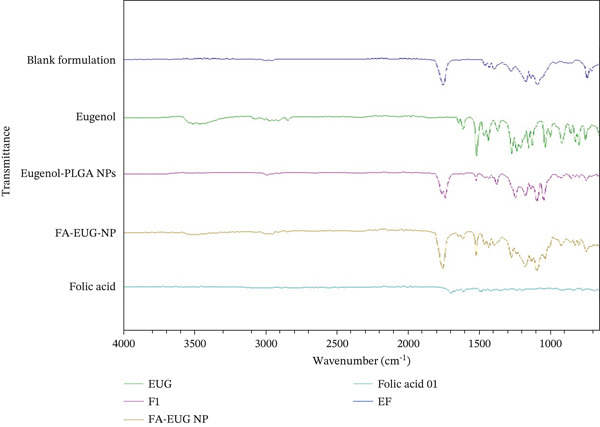
FTIR analysis of blank formulation (without drug), pure drug eugenol, eugenol‐loaded PLGA NPs, folic acid, and FA‐conjugated eugenol‐loaded PLGA NPs.

FTIR analysis was carried out to confirm the compatibility between the pure eugenol, FA, eugenol‐loaded PLGA NPs, FA‐conjugated eugenol‐loaded NPs, and blank formulation (without drug), as shown in Figure 3. The results of FTIR data revealed that there was no major shifting as well as no loss of functional peaks between the pure drug eugenol, eugenol‐loaded PLGA NPs, FA, and FA‐conjugated eugenol‐loaded PLGA NPs. The spectrum of FA‐conjugated eugenol‐loaded PLGA NPs exhibited peaks that signify the conjugation of FA and eugenol (1491 and 1037 cm^−1^).

**Figure 4:**
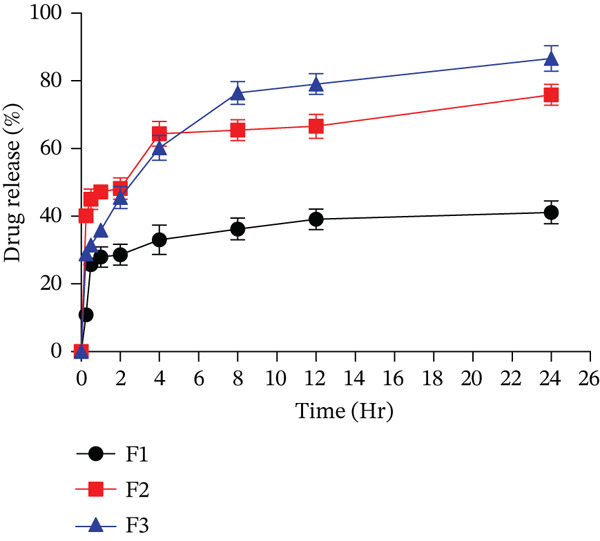
In vitro drug release study of FA‐conjugated eugenol‐loaded PLGA NPs. Values (*n* = 3) are mean ± SD.

The in vitro study of different formulations of FA‐conjugated eugenol‐loaded PLGA NPs was performed by the dialysis bag membrane method. The results of the *F*1 formulation with a 1:1 ratio of drug and polymer showed a release of 41.41*%* ± 3.39*%* in 24 h, the *F*2 formulation 1:2 of drug and polymer ratio showed a release of 75.88*%* ± 3.08*%* in 24 h, and the *F*3 formulation 1:3 drug and polymer ratio showed a release of 86.63*%* ± 3.76*%* in 24 h, as shown in Figure 4. The *F*3 formulation showed the highest release of the drug in 24 h. It is observed that the *F*3 formulation showed faster drug release for the first 8 h as compared to the *F*1 and *F*2 formulations.

Data collected from in vitro drug release studies were employed to analyze release kinetics, as illustrated in Table 4. The release kinetics were determined by fitting the release data to various models, including zero‐order, first‐order, Higuchi, Korsmeyer–Peppas, and Hixon–Crowell models. The data integrity was assessed using the regression factor. The in vitro drug release profiles of formulations were most suitably described by the Higuchi kinetic model, suggesting that drug release is predominantly controlled by diffusion of the drug from the polymer matrix. Formulation *F*3 showed the highest *R* ^2^ value, which was close to 0.95, indicating that the release of the drug from the NP formulation followed the Higuchi model.

**Table 4:** In vitro drug release kinetics of different formulations of FA‐conjugated eugenol NPs.

Formulation	Zero‐order		First‐order		Higuchi		Korsmeyer–Peppas			Hixon–Crowell	
	*K*_0_	*R* ^2^	*K*_1_	*R* ^2^	*K*_*H*_	*R* ^2^	*K*_*K* *P*_	*n*	*R* ^2^	*K*_*H* *C*_	*R* ^2^
F1	32.71	0.46	1.81	0.62	21.32	0.70	42.61	0.14	0.25	15.98	0.13
F2	16.09	0.48	1.92	0.54	9.15	0.73	21.10	0.17	0.41	15.65	0.12
F3	22.81	0.79	1.89	0.94	9.83	0.95	35.25	0.26	0.54	15.75	0.15

**Figure 5:**
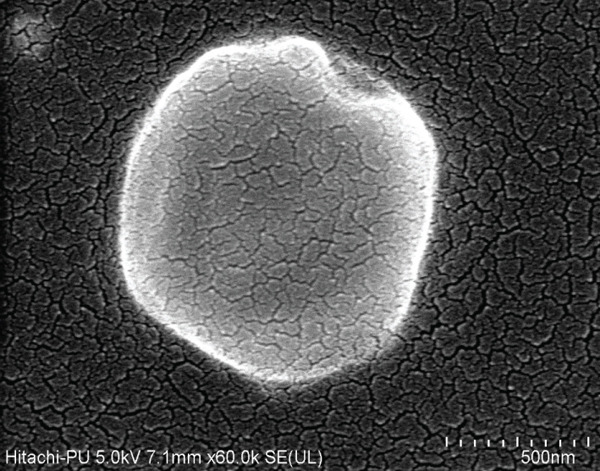
Morphology characterization of FA‐conjugated eugenol‐loaded PLGA NPs analyzed by FESEM analysis.

Formulation F3 was selected for further evaluation, considering the outcomes of particle size analysis, PDI, zeta potential measurements, and in vitro drug release studies. The morphological characteristics of the FA‐conjugated eugenol‐loaded PLGA NPs were analyzed by FESEM analysis, and the results are shown in Figure 5. The particles were found to be in the nanorange with spherical to elliptical shapes with fine cracks on the surface.

**Figure 6:**
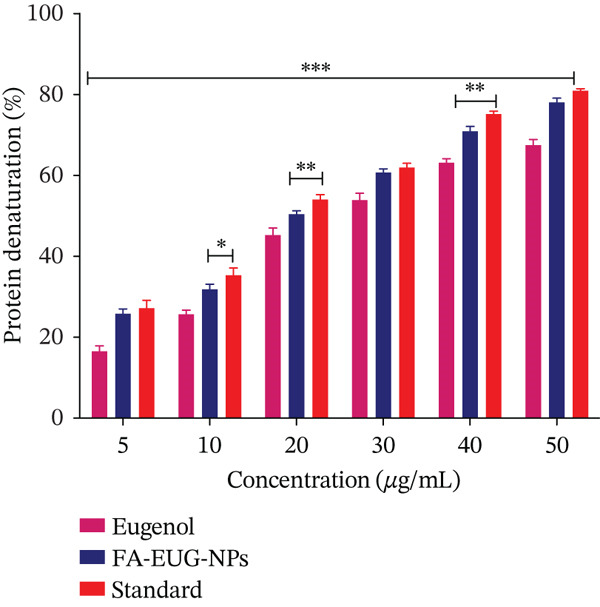
Anti‐inflammatory activity of eugenol, FA‐conjugated eugenol‐loaded PLGA NPs, and standard (diclofenac sodium). Values are analyzed by ANOVA analysis. ∗∗∗ represents a significant difference with *p* value < 0.001, ∗∗ represents *p* value < 0.01, and ∗ represents *p* value < 0.05 when compared with pure drug eugenol. Values (*n* = 3) are mean ± SD.

FA‐conjugated eugenol‐loaded PLGA NPs showed an inhibition of 78.05*%* ± 1.02*%* and an IC_50_ value of 23.74 *μ*g/mL, whereas pure drug eugenol showed 67.43*%* ± 1.43*%* and an IC_50_ value of 28.83 *μ*g/mL. Diclofenac sodium (5–50 *μ*g/mL) was used as a reference drug, and it showed inhibition of protein denaturation of 80.90*%* ± 0.52*%* and an IC_50_ value of 21.07 *μ*g/mL. The vehicle control, DMSO, had a negligible effect on protein denaturation. FA‐conjugated NPs showed more anti‐inflammatory activity as compared to pure drug eugenol, as shown in Figure 6. These results suggest that FA‐conjugated eugenol‐loaded PLGA NPs possess significant anti‐inflammatory activity.

**Figure 7:**
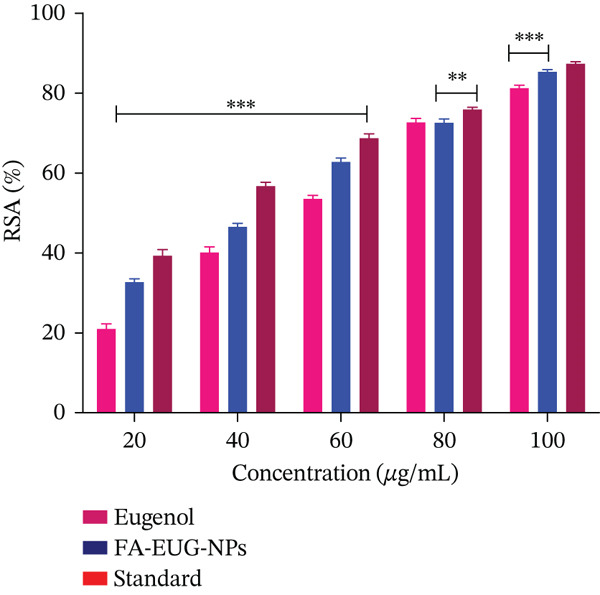
Antioxidant activity of eugenol, FA‐conjugated eugenol‐loaded PLGA NPs, and standard ascorbic acid. Values are analyzed by ANOVA analysis. ∗∗∗ represents a significant difference with *p* value < 0.001, and ∗∗ represents *p* value < 0.01 when compared with pure drug eugenol. Values (*n* = 3) are mean ± SD.

The free radical scavenging activities of the pure drug eugenol and FA‐conjugated eugenol‐loaded PLGA NPs were estimated by DPPH assay. The eugenol showed a percentage inhibition of 81.20*%* ± 0.759*%*, and the IC_50_ value was 55.18 *μ*g/mL, whereas FA‐conjugated eugenol‐loaded PLGA NPs showed a percentage inhibition of 85.28*%* ± 0.984*%*, and the IC_50_ value was 45.34 *μ*g/mL. Ascorbic acid, which is taken as a positive control, showed a percentage inhibition of 87.13*%* ± 0.508*%*, and the IC_50_ value was 25.13 *μ*g/mL, as shown in Figure 7. The vehicle control methanol showed no significant effect on DPPH radicals. The lower the IC_50_ value, the greater the antioxidant potential. These results suggest that FA‐conjugated eugenol‐loaded PLGA NPs possess significant antioxidant activity.

**Figure 8:**
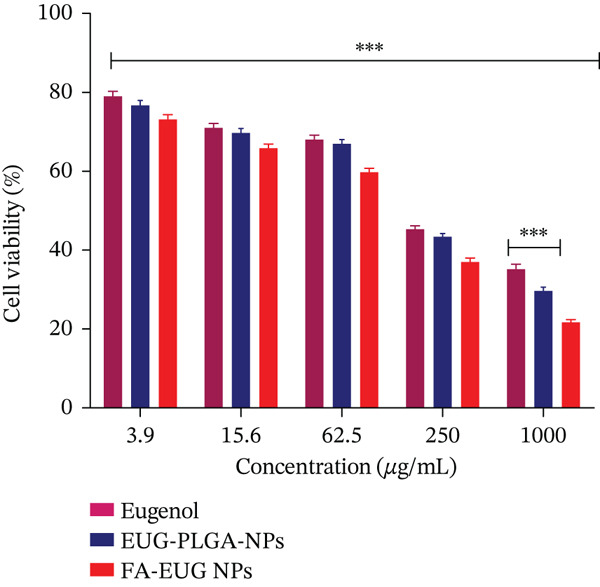
In vitro evaluation of the cytotoxicity on MDA‐MB‐231 cancer cell lines; values are analyzed by ANOVA analysis (*p* < 0.001). ∗∗∗ represents a significant difference with *p* value < 0.001 when compared with pure drug eugenol. Values (*n* = 3) are mean ± SD.

The MDA‐MB‐231 cell line (BC cell line) was used to check the effect of eugenol, eugenol‐loaded PLGA NPs, and FA‐conjugated eugenol‐loaded PLGA NPs. The pure eugenol drug exhibited a notable cytotoxic impact on the cell line, reducing their viability across various concentrations (3.9–1000 *μ*g/mL). The cell viability (percentage) of FA‐conjugated eugenol‐loaded PLGA NPs at the highest concentration was found to be 21.70*%* ± 0.697*%*, eugenol‐loaded PLGA NPs showed cell viability of 29.68*%* ± 0.94*%*, and eugenol showed cell viability of 35.30*%* ± 1.14*%*. Eugenol exhibited an IC_50_ value of 519.42 *μ*g/mL, eugenol‐loaded PLGA NPs exhibited an IC_50_ value of 438.01 *μ*g/mL, whereas FA‐conjugated eugenol‐loaded PLGA NPs exhibited an IC_50_ value of 298.81 *μ*g/mL. The vehicle control, DMSO, had a negligible effect on cell viability. The results showed that FA‐conjugated Eugenol‐loaded PLGA NPs showed the least cell viability as compared to the eugenol‐loaded PLGA NPs and pure drug eugenol, as shown in Figure 8. FA‐conjugated NPs exhibited a lower IC_50_ value as compared to eugenol‐loaded PLGA NPs and eugenol. These results suggest that FA‐conjugated eugenol‐loaded PLGA NPs possess significant cytotoxic activity against MDA‐MB‐231 cells.

## 4. Discussion

The pure compound eugenol was obtained through the column chromatography technique and was characterized by TLC, FTIR, ^1^H NMR, ^13^C NMR, and HPTLC. Eugenol‐loaded PLGA NPs were synthesized by the modified solvent‐emulsification diffusion technique. The isolated and characterized eugenol was used in our further studies to prepare its polymeric NPs and conjugated with FA and its in vitro assessment for antioxidant, anti‐inflammatory, and cytotoxicity properties.

The *F*3 formulation drug:polymer ratio (1:3) showed the highest entrapment of drug among all three different formulations. This could be due to the increased ratio of polymer in the formulation. With an increased PLGA polymer ratio, there is more polymer available to encapsulate eugenol. This higher polymer concentration provides more opportunities for the drug molecules to be encapsulated within the polymer matrix, leading to a higher EE% [47].

The size of particles and their distribution significantly influence the performance of NPs. Batches displaying a broad particle size distribution can cause notable variations in drug release, bioavailability, and efficacy. Particle size is the most important factor because it has a direct relevance to the stability of the NPs [48, 49]. The polymer′s terminal carboxyl group presence resulted in higher negative values of zeta potential for the formulated NPs. In the case of charged particles, the repulsive contact will get stronger as the zeta potential rises, resulting in the production of more stable particles with a more consistent size distribution. Lowering the negative zeta potential results in increased stability of NPs. These findings suggest that the drug was not absorbed onto the NPs′ surface [47].

The lower FA conjugation percentage in formulation *F*3 primarily resulted from the masking of FA binding sites on NPs. This was due to the higher encapsulation percentage of eugenol in the carrier, along with possible availability on the particle surface [47].

The results of FTIR data revealed that there are no major shifts as well as no loss of functional peaks between the pure drug eugenol, eugenol‐loaded PLGA NPs, FA, and FA‐conjugated eugenol‐loaded PLGA NPs. The spectrum of FA‐conjugated eugenol‐loaded PLGA NPs exhibited peaks that signify the conjugation of FA and eugenol. Although FTIR analysis provided initial evidence of conjugation of FA and eugenol, it is acknowledged that this method has limitations in terms of providing detailed information on the surface modification. Future studies would benefit from employing advanced techniques such as XPS and NMR to further confirm the successful conjugation of FA. These techniques would provide a more robust and detailed characterization of the NP surface, enabling a better understanding of the conjugation process and its impact on NP properties.

The in vitro study of different formulations of FA‐conjugated eugenol‐loaded PLGA NPs was performed by the dialysis bag membrane method. It is observed that the *F*3 formulation showed faster drug release for the first 8 h as compared to *F*1 and *F*2 formulations. This may be due to higher drug loading and faster drug diffusion from the outer layers of the polymeric particles. Each sample demonstrated a biphasic release profile, characterized by an initial phase of rapid release succeeded by a sustained release maintained at a consistent rate. This observed trend might be attributed to the surface binding of eugenol to the carrier [50, 51].

The in vitro drug release profiles of the formulations were analyzed by fitting the release data to various kinetic models, including zero‐order, first‐order, Higuchi, Korsmeyer–Peppas, and Hixon–Crowell. Among these, the Higuchi model provided the best fit, indicating that the release of the drug was primarily controlled by diffusion from the polymer matrix. The *F*3 formulation, in particular, exhibited the highest *R* ^2^ value (0.95), supporting the hypothesis that its drug release follows the Higuchi model. This result is consistent with previous studies of diffusion‐driven release mechanisms in matrix‐based systems [39, 40, 52], suggesting the Higuchi model is well suited for describing the release kinetics of NP formulations for controlled drug delivery.

FESEM analysis showed that particles were found to be in the nanorange with spherical to elliptical shapes with fine cracks on the surface. These features are particularly significant in cancer studies, as NPs in the nanometer range are known to enhance cellular uptake due to their small size, allowing for efficient drug delivery and tumor penetration [53, 54].

FA‐conjugated eugenol NPs might exhibit enhanced protein denaturation activity compared to pure eugenol due to their increased surface area, improved stability, and controlled release properties. The smaller size of NPs allows them to interact more effectively with proteins, leading to greater denaturation activity [55].

On the basis of free radical scavenging activity, the IC_50_ value of FA‐conjugated eugenol‐loaded PLGA NPs was significantly lower (*p* < 0.001) than the IC_50_ value of pure eugenol. This is due to the reason that NPs can protect the drug from degradation and enhance its stability, ensuring a sustained release of antioxidants and prolonged activity [56].

BC remains one of the most lethal malignancies affecting women worldwide. While substantial advances have been made in the treatment of BC over recent decades [57], improvements in patient survival rates and living standards have been limited. One of the primary challenges associated with BC chemotherapy is the occurrence of severe side effects induced by synthetic cytotoxic agents, in addition to the development of drug resistance after repeated treatment cycles. To address these issues, targeted drug delivery systems that utilize naturally derived bioactive compounds, such as eugenol, encapsulated in biocompatible carrier material, offer a promising solution. These systems have the potential to alleviate the adverse effects of traditional chemotherapy. The cytotoxicity of pure eugenol, eugenol‐loaded PLGA NPs, and FA‐conjugated eugenol‐loaded PLGA NPs was assessed on MDA‐MB‐231 BC cells. The metabolic activity of the treated cells was measured using the MTT assay. The results showed that FA‐conjugated eugenol‐loaded PLGA NPs showed the least cell viability as compared to the eugenol‐loaded PLGA NPs and pure drug eugenol, as shown in Figure 8. FA‐conjugated NPs exhibited a lower IC_50_ value as compared to eugenol‐loaded PLGA NPs and eugenol. This decline primarily arises from the targeted effect of FA and enhanced drug release from the hybrid biocompatible carrier post FA‐mediated endocytosis of the carrier system [47]. The drug encapsulation within the carrier system facilitated its controlled release. Therefore, the formulated system demonstrates significant potential for targeted chemotherapy, effectively inhibiting tumor growth, progression, and metastasis. The enhanced cytotoxicity of FA‐conjugated eugenol‐loaded PLGA NPs in MDA‐MB‐231 cells is consistent with previous studies demonstrating the effectiveness of folate receptor‐targeted NPs in delivering therapeutic agents to BC cells [21]. It has been reported that folate‐conjugated liposomes showed increased uptake in folate receptor‐positive cancer cells [21]. Similarly, it has been demonstrated that folate‐targeted NPs enhanced the delivery of anticancer agents to BC cells, resulting in improved therapeutic outcomes. Furthermore, the cytotoxic effects of eugenol against BC cells have been documented [15]. Our results support these findings and suggest that FA‐conjugated eugenol‐loaded PLGA NPs may be a promising approach for BC treatment.

## 5. Conclusion

The study illustrates the isolation of pure compound eugenol from *S. aromaticum* bud and the synthesis of polymeric NPs from the bioactive fraction (pure compound). In the present work, FA‐conjugated eugenol‐loaded PLGA NPs were developed specifically for targeting the delivery of natural cytotoxic agents to BC cells. An amplified bioactivity was noted in the eugenol‐loaded PLGA NPs. These NPs exhibited an increased drug encapsulation efficiency of 87.51% and displayed improved eugenol release characteristics, potentially advantageous for cancer treatment applications. The results demonstrated higher in vitro antioxidant and higher in vitro anti‐inflammatory activity of FA‐conjugated eugenol‐loaded PLGA NPs. In the in vitro cell cytotoxicity assay conducted on MDA‐MB‐231 cells, it was observed that FA‐conjugated NPs exhibited a greater capacity to target FA receptor‐positive BC cells compared to nonconjugated eugenol‐loaded PLGA NPs. As a conclusion, the formulated eugenol‐loaded PLGA NPs demonstrate potential suitability as a carrier platform for the targeted delivery of eugenol in BC treatment. This study introduces a novel FA‐conjugated PLGA NP system designed for the targeted delivery of eugenol, which has not been extensively explored. The developed FA‐conjugated eugenol‐loaded PLGA NPs exhibit significantly higher encapsulation efficiency, sustained drug release, and improved bioactivity. The FA conjugation enhances selective uptake by FA receptor‐positive BC cells, making this formulation a promising alternative to conventional chemotherapy. By integrating nanotechnology with natural phytochemicals, this study provides a new therapeutic approach for BC treatment and bridges the gap between natural compound‐based therapy and advanced nanomedicine.

## 6. Limitations and Future Recommendations

This study demonstrates the potential of FA‐conjugated eugenol‐loaded PLGA NPs, but several limitations need to be addressed. The first limitation is that the cell viability experiments did not include blank NPs as a control. Including blank NPs in future assays would allow researchers to separate the drug′s specific activity from any influence of the delivery system. The lack of in vivo studies hinders the understanding of pharmacokinetics and biodistribution and tumor targeting, which are essential for validating the safety and efficacy of these NPs in human systems. Further studies are required to evaluate the efficacy of FA‐conjugated eugenol‐loaded PLGA NPs in vivo, assessing tumor targeting and systemic toxicity in animal models. Future in vivo studies using the MDA‐MB‐231 xenograft model should focus on optimizing NP stability, bioavailability, and tumor accumulation while minimizing immune clearance to enhance the therapeutic efficacy of FA‐conjugated eugenol‐loaded PLGA NPs. Another limitation related to stability and bioavailability has not been addressed. Future research should focus on these limitations to enhance the clinical applicability of these NPs.

FA‐conjugated eugenol‐loaded PLGA NPs hold tremendous promise for future clinical and industrial applications, particularly in targeted cancer therapy. It can revolutionize BC treatment by specifically targeting cancer cells, enhancing therapeutic efficacy through eugenol′s antioxidant and cytotoxic properties. Industrially, it can drive nanotechnology‐based cancer treatment innovation, scalability, and commercialization.

## Author Contributions

B.K. has done the investigation and drafted the manuscript. A.K. has conceptualized and supervised. T.M. has edited and validated the results. D.N.K. has conceptualized, validated the results, analyzed, and supervised.

## Funding

No funding was received for this manuscript.

## Conflicts of Interest

The authors declare no conflicts of interest.

## Supporting information


**Supporting Information** Additional supporting information can be found online in the Supporting Information section. Figure S1: FTIR analysis of pure isolated compound. Figure S2: NMR spectra of pure isolated compound eugenol (A) ^1^H NMR and (B) ^13^C NMR. Figure S3: HPTLC analysis of isolated pure compound eugenol.

## Data Availability

The datasets used and/or analyzed during the current study are available from the corresponding authors upon reasonable request.
