# Supplementary material for: Folic Acid–Conjugated PLGA Nanoparticles of Eugenol: Development, Characterization, and In Vitro Cytotoxicity Studies on Breast Cancer Cell Line
Source: Biomed Res Int. 2026 Apr 27;2026:2898012. doi: 10.1155/bmri/2898012 (PMC13111985; doi:10.1155/bmri/2898012)
Supplement: Supplementary file 1 — Supporting Information Additional supporting information can be found online in the Supporting Information section. Figure S1: FTIR analysis of pure isolated compound. Figure S2: NMR spectra of pure isolated compound eugenol (A) 1H NMR and (B) 13C NMR. Figure S3: HPTLC analysis of isolated pure compound eugenol. [file BMRI-2026-2898012-s001.docx]

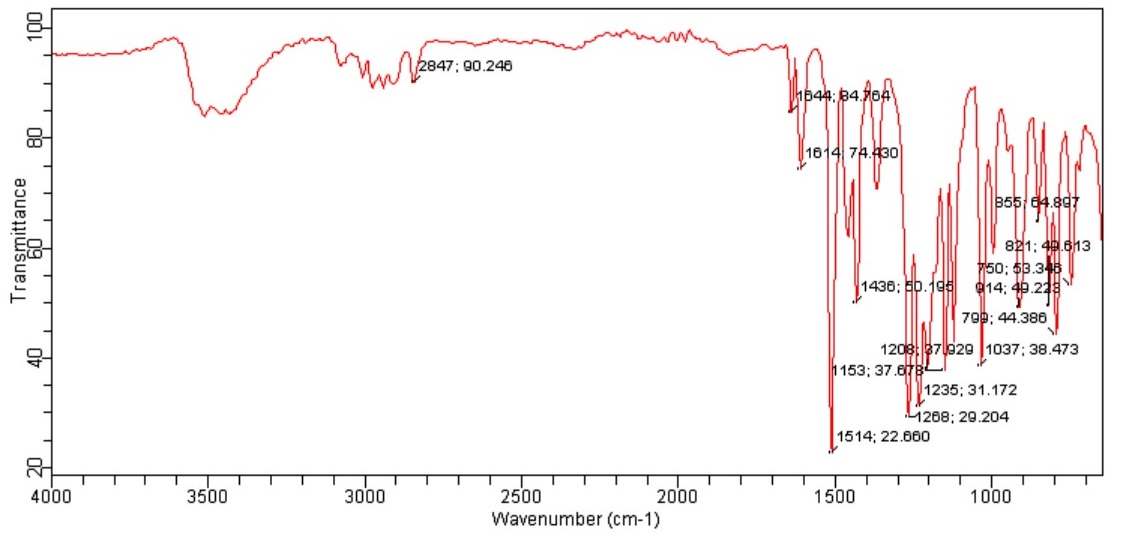


**Figure S1:** FTIR analysis of pure isolated compound.


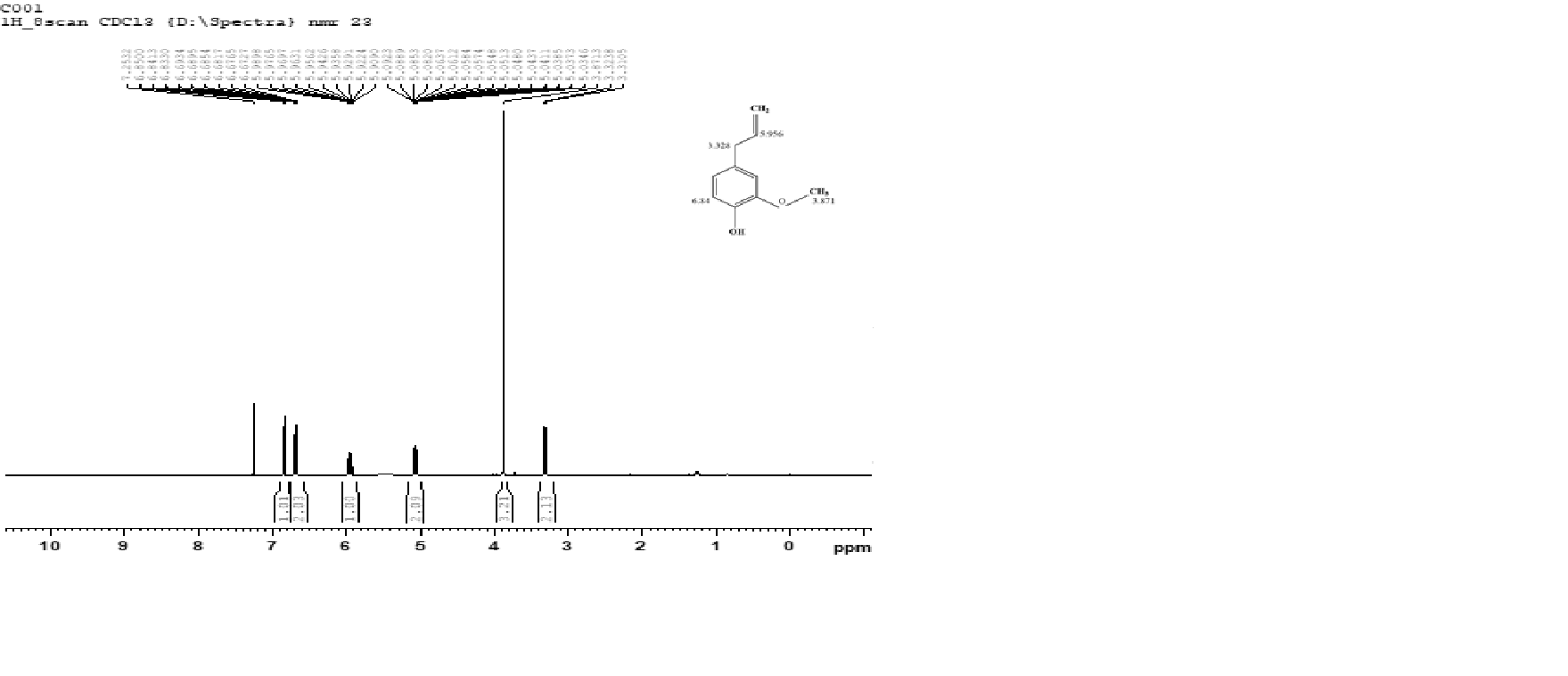


**(A)**

**
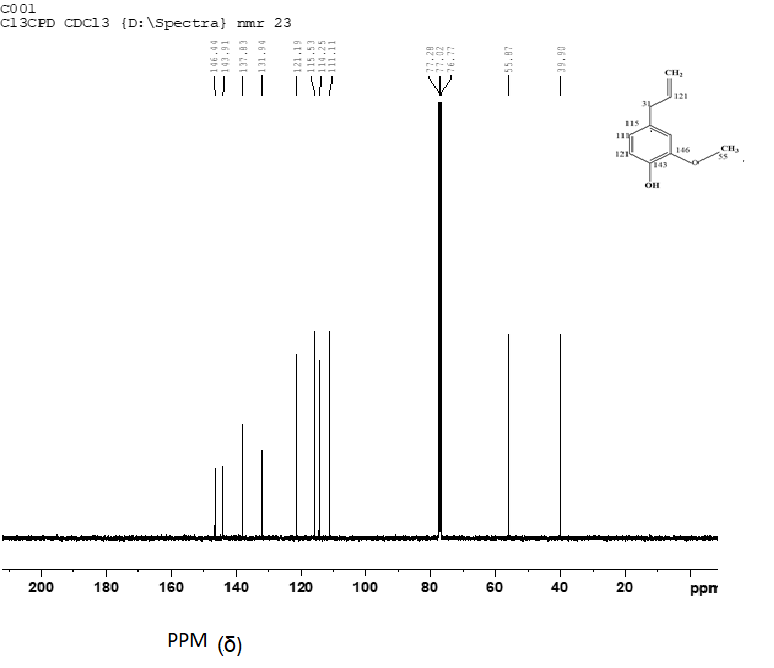
**

**(B)**

**Figure S2:** NMR spectra's of pure isolated compound Eugenol S2(A) ^1^H-NMR and S2(B) ^13^C-NMR.


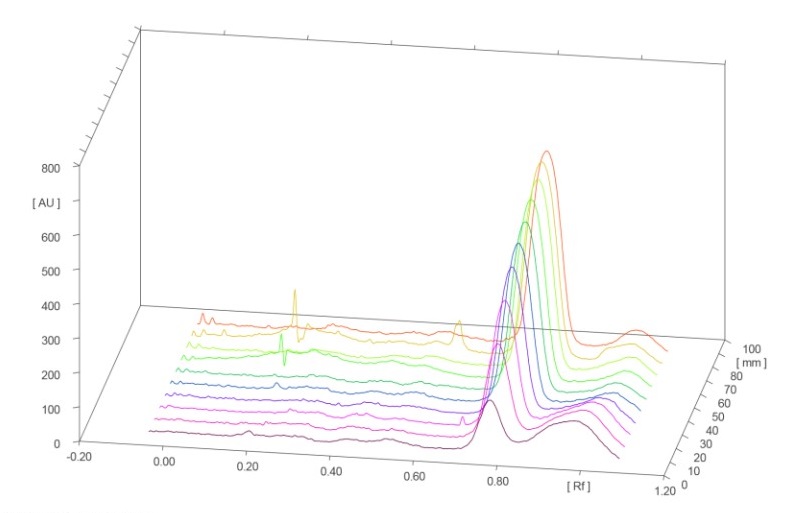


**Figure S3:** HPTLC analysis of isolated pure compound Eugenol.
